# Foreign

**Published:** 1841-11-27

**Authors:** 


### FOREIGN.

*On the Radical Cure of Spina Bifida by a New Operation.* By M. Dubourg, Physician to the Hospital of Marmande.—Having observed that the ordinary method of treating spina bifida, was far from successful, and reflecting that when the soft parts over bones separated by arrest of development are brought together by suture, they have considerable effect in approximating the edges of bone, as in cleft palate, M. Dubourg concluded that by bringing together the soft parts over the defective spot in the spinal column, a radical cure might be effected by producing permanent approximation of the edges of the bony opening. The first case in which he put his ideas into execution proved unsuccessful, the child having died two days afterwards, but in two other cases the success was complete.

Case i. In the spring of 1837 I was called to a female infant eight days old, who had a congenital pediculated tumour on the lumbar region, about the size of an apple. Its colour was livid from the development of a venous network, giving it the appearance of a vascular fungus. It was evident on examining the vertebræ that they were defectively formed, the edges of a bony opening being discovered. The tumour was opaque, its walls much thicker than usual, and it appeared that the arrest of development was confined to the last lumbar vertebræ, the child being otherwise healthy and well formed. Having the cautery in readiness in case of severe bleeding from the vascular coverings, and being prepared to prevent the sudden escape of the spinal fluid, an elliptical incision was made around the base of the tumour, when a quantity of reddish serum escaped, and the excision of the sac was readily effected. The finger passed readily into the spinal canal. The edges of the wound were brought together, and four needles being passed, the twisted suture was applied as in a case of hare-lip. The threads were twisted so as to exert as much traction as possible on the contiguous parts, and small compresses were placed at the extremities of the needles to protect the skin. The child cried sharply at the commencement of the operation, but as soon as the fluid escaped from the spinal canal it fell for a few minutes into a state of stupor. It cried again as the needles were applied, and by the time the dressing was completed it took the breast as though nothing had occurred. The needles and sutures were removed on the fourth day, when the edges of the wound were found to be united. Adhesive plaster was applied, and in fifteen days a strong cicatrix, forming a sort of solid button filling up the opening in the vertebræ, was all that remained of this reputed incurable disease. The examination of the removed tumour demonstrated that it was a cyst distended by fluid, communicating with the spinal marrow, bounded behind by the common integument and an expansion of the arachnoid and dura mater. The cavity was not in proportion with the volume of the tumour, for there were several layers of the cellular and adipose tissue between its external and internal surfaces.

Case ii. The next case was similar, except that the swelling was situated over the junction of the last cervical and first dorsal vertebræ. The same operation was performed except that the sac was not perforated in the first incision; a lateral flap being formed with a narrow bistoury from within outwards, and the sac being then removed and the other flap formed by a second incision. The needles and sutures were applied as soon as possible to prevent the loss of any considerable quantity of spinal fluid, and the access of air. The child recovered without an unfavorable symptom and continues well, eighteen months after operation.

The following are the conclusions drawn from these cases by the author, some of which, at least, are premature:

1. Some cases of spina bifida are susceptible of a radical cure.

2. Instead of abandoning to their fate most children born with this malformation, those should be selected in which this operation might be efficacious.

3. Although it is impossible absolutely to establish limits of incurability, every child born with a spina bifida, of which the opening of communication with the spine does not exceed an inch in diameter should be submitted to operation.

4. Ablation of the tumour and the twisted suture constitute the best operation.

5. It is well to open the spinal canal as late as possible in the operation, and to make the opening narrow.

6. When the sac is formed by the meninges, only the skin being atrophied over the tumour, the skin must be dissected on each side of the vertebræ, and the edges of the integuments united as in hare-lip.

7. The probability of reunion is proportionate to the size of the osseous opening and the general state of the patient.

8. The spinal canal may be opened, the spinal marrow laid bare, and a considerable portion of the fluid which bathes this important organ lost with impunity.—*Brit. and For. Med. Rev.*, *from Gazette Medicale de Paris*, *Juliet* 31, 1841.

---

*On the Chorda Dorsalis.* By Martin Barry, M.D., F.R.S., L. & E.—The author of this communication, after pointing out the similarity in appearance between an object noticed by him in the mammiferous ovum, and the incipient chorda dorsalis described by preceding observers in the ova of other vertebrata, mentions some essential differences between his own observations and those of others, as to the nature and mode of origin of these objects, and their relation to surrounding parts. Von Baer, the discoverer of the chorda dorsalis, describes this structure as "the axis around which the first parts of the fœtus form." Reichert supposes it to be that embryonic structure which serves as "a support and stay" for parts developed in two halves. The author's observations induce him to believe that, instead of being "the axis *around which* the first parts of the fœtus form," the incipient chorda is the last-formed row of cells, which have pushed previously-formed cells farther out; and that, instead of being merely "a support and stay" for parts developed in two halves, the incipient chorda occupies the centre out of which the "two halves" originally proceeded as a single structure, and is itself in the course of being enlarged by the continued origin of fresh substance in its most internal part.

The author enters into a minute comparison of the objects in question, from which it appears that the incipient chorda is not, as Baer supposed, developed into a globular form at the fore end, but that the linear part is a process from the globular, and that the pellucid cavity contained within the latter—a part of prime importance, being the main centre for the origin of new substance—is not mentioned by Von Baer. Farther, that the origin of the "laminæ dorsales" of this naturalist (the "central nervous system" of Reichert) is not simultaneous with, but anterior to, that of the chorda.

The author then reviews the observations of Rathke and Reichert on the chorda dorsalis, which contain internal evidence, he thinks, of a process in the development of fishes, reptiles and birds, the same as that which he has observed in mammalia, namely, the origin of the embryo out of the nucleus of a cell.

And it is his opinion that this observation may assist to solve a question on which physiologists are not agreed; for it shows that if the nucleus of a cell is a single object, the first rudiments of the embryo are not two halves. The author thinks that unless the very earliest periods are investigated, it is in vain that we attempt to learn what that is of which the rudiments of the embryo are composed. From not attending to this, physiologists have supposed their "primitive trace" to arise in the substance of a membrane, which the author, in his second series on the embryo, showed could not be the case. To the same cause, he thinks, is referrible an opinion recently advanced by Reichert, that the first traces of the new being are derived from cells of the yolk.—*Ib., from Proceedings of the Royal Society.* 1841.

